# Editorial: Advances of neurobiological basis and psychopathological mechanism for mood disorders

**DOI:** 10.3389/fpsyt.2023.1333775

**Published:** 2023-11-27

**Authors:** Yue Dong, Yuankang Liu, Chengqi Xu, Ranji Cui, Yao-Ying Ma, Hua Wei, Xiangyang Xu

**Affiliations:** ^1^Department of Radiology, Liyuan Hospital, Tongji Medical College, Huazhong University of Science and Technology, Wuhan, China; ^2^Key Laboratory of Molecular Biophysics of the Ministry of Education, College of Life Science and Technology and Center for Human Genome Research, Huazhong University of Science and Technology, Wuhan, China; ^3^Second Affiliated Hospital of Jilin University, Changchun, China; ^4^School of Medicine, Indiana University Bloomington, Indianapolis, IN, United States; ^5^Normal College, Qingdao University, Qingdao, Shandong, China

**Keywords:** mood disorders, psychopathological mechanisms, heart rate variability, neurobiological, biomarkers

Mental disorders are a group of diseases that are characterized by cognitive, emotional, and behavioral disturbances, affecting over 450 million people worldwide (1). They not only seriously threaten people's health and lives but also impose negative social and economic consequences (2). Mood disorders are one of the most common types of mental disorders in the world, and their prevalence continues to rise, particularly during the COVID-19 pandemic (3). However, the neurobiological basis and psychopathological mechanisms of mood disorders have not been fully explained, which limits the establishment of therapeutic strategies. Therefore, in this Research Topic, we focused on the latest advancements in the neurobiological basis, psychopathological mechanism, and diagnostic strategies of mood disorder, which may explain the cause of mood disorders and provide a theoretical basis for clinical interventions.

Deficits in attentional control are an important factor influencing depressive mood development. Heart rate variability (HRV) is considered to be a psychophysiological marker, which is regulated by the interaction of the vagal and sympathetic components of the autonomic nervous system and indirectly, objectively, and reliably reflects the functioning of the autonomic nervous system (4). It is defined as the variability of the interval between successive heartbeats, and the measurement is feasible and reliable. Zheng et al. conducted a cross-sectional study with 220 college students to verify whether gender moderates the relationship between attentional control and resting vaguely mediated HRV (vmHRV). Results found that women have worse attentional shifting abilities and higher resting vmHRV than men. Attentional shifting facilitates the way people shift their attention away from negative thoughts and information. Higher resting vmHRV in women reflects a compensatory response to deficits in attentional control. These results help explain why it is easier for women to become depressed and provide neurophysiological evidence to elucidate gender differences in attentional control.

Psychophysiological biomarkers are considered more objective metrics in the diagnosis of mood disorders. Ham et al. examined the link between the self-rated questionnaire and the clinician-rated assessments, and HRV indices in patients with depressive and anxious symptoms. They observed that HRV variables showed significant associations only with the clinician-rated assessments. Furthermore, a stronger relationship was observed between depression and HRV indices; the severity of depressive symptoms could be objectively assessed through the HRV indices.

Recently, neuroimaging has emerged as a valuable method for investigating the pathogenesis of mood disorders. Functional MRI (fMRI) based on blood oxygenation level-dependent signals fractional amplitude of low-frequency fluctuation (fALFF) can be used to detect neural activity during the resting state (5). Zhang et al. conducted a study to investigate the influence of sleep disturbances (SD) on clinical characteristics of major depressive disorder (MDD) patients, using resting-state fMRI (rs-fMRI). They observed that both MDD patients without SD symptoms (Pa_s) and patients with SD symptoms (Pa_ns) displayed abnormal fALFF in the frontal-occipital brain regions, suggesting that this is a common characteristic of MDD patients. In addition, the author found Pa_s showed a higher fALFF value in the left precuneus compared with Pa_ns, which could be used to distinguish MDD patients with or without SD symptoms. These results suggest a higher fALFF value in the left precuneus could be a specific neuroimaging characteristic differentiating the two groups in clinical practice. However, due to the small sample size, these results should be interpreted with caution and need to be validated in distinct SD performance.

Collectively, the articles collected on this topic included studies on neurophysiological mechanisms and psychophysiological and early imaging change markers for mood disorders. Recently, new promising technologies have been emerging, especially artificial intelligence and genomics. Future studies should continue to discover more specific biomarkers that may expand the understanding of mood disorders and examine the effects of interventions targeting these biomarkers for mood disorders.

## Author contributions

YD: Writing—original draft. YL: Writing—original draft. CX: Supervision, Validation, Writing—review & editing. RC: Writing—review & editing. Y-YM: Writing—review & editing. HW: Writing—review & editing. XX: Supervision, Validation, Writing—review & editing.
